# What helps or hinders effective end-of-life care in adult intensive care units in Middle Eastern countries? A systematic review

**DOI:** 10.1186/s12904-024-01413-7

**Published:** 2024-04-01

**Authors:** Nabat Almalki, Breidge Boyle, Peter O’Halloran

**Affiliations:** 1 Prince Sultan Military College for Health Sciences, Dharan, Saudi Arabia; 2 School of Nursing and Midwifery, Queen’s University Belfast, Medical Biology Centre, 97 Lisburn Road, Belfast, BT9 7BL UK

**Keywords:** End-of-life care, Intensive care unit, Terminal illness, Challenges, Facilitators, Middle Eastern countries

## Abstract

**Background:**

As many patients are spending their last days in critical care units, it is essential that they receive appropriate end-of -life care. However, cultural differences, ethical dilemmas and preference practices can arise in the intensive care settings during the end of life. Limiting therapy for dying patients in intensive care is a new concept with no legal definition and therefore there may be confusion in interpreting the terms ‘no resuscitation’ and ‘comfort care’ among physicians in Middle East. Therefore, the research question is ‘What helps or hinders effective end-of-life care in adult intensive care units in Middle Eastern countries?’

**Methods:**

The authors conducted a comprehensive systematic literature review using five electronic databases. We identified primary studies from Medline, Embase, CINAHL, Psycinfo and Scopus. The team assessed the full-text papers included in the review for quality using the Joanna Briggs Institute checklist (JBI). We completed the literature search on the first of April 2022 and was not limited to a specific period.

**Results:**

We identified and included nine relevant studies in the review. We identified five main themes as end-of-life care challenges and/or facilitators: organisational structure and management, (mis)understanding of end-of-life care, spirituality and religious practices for the dying, communication about end-of-life care, and the impact of the ICU environment.

**Conclusions:**

This review has reported challenges and facilitators to providing end-of-life care in ICU and made initial recommendations for improving practice. These are certainly not unique to the Middle East but can be found throughout the international literature. However, the cultural context of Middle East and North Africa countries gives these areas of practice special challenges and opportunities. Further observational research is recommended to confirm or modify the results of this review, and with a view to developing and evaluating comprehensive interventions to promote end-of-life care in ICUs in the Middle East.

## Introduction

Globally, the majority of people with chronic diseases such as cardiovascular diseases, chronic respiratory diseases, kidney failure, and cancer require special care at the end-of-life (EOL) phase when their health worsens before death [1, 2]. As the severity of illness of hospitalised patients increases, advanced technological support has increasingly helped such patients to survive longer [3, 4]. However, the focus on technology in intensive care units (ICUs) can make the implementation of palliative and end-of-life care more problematic [5]. This is because the ICU is typically a curative setting that aims to treat reversible causes of serious illness [5, 6].

The World Health Organisation defines palliative care as an approach that improves the quality of life of patients (adults and children) and their families who are facing problems associated with life-threatening illness. It prevents and relieves suffering through the early identification, correct assessment and treatment of pain and other problems, whether physical, psychosocial or spiritual [7]. According to The National Hospice and Palliative Care Organisation end-of-life care commences when an individual has a diagnosis of a terminal illness with less than six months to live, and curative therapies are no longer options [8]. Whilst there is an emerging international consensus on core goals and principles for palliative and EOL care in the ICU, there remain many differences in the outworking and application of these principles, depending on the legal, cultural and religious context for clinical practice [9]. This is especially the case for issues relating to withholding and withdrawing life-sustaining treatment, while ensuring the alleviation of suffering [10].

Nevertheless, it is widely accepted that providing aggressive care may not always benefit critically ill patients, and if curative care proves unsuccessful a change in management goals from restorative care to palliative care will be considered with patient and families. However, according to many ICU healthcare providers this transition is the most complicated stage in providing end-of-life care [11, 12], and discussion about comfort and EOL goals is often postponed until it is obvious that death is near [13].

For the purposes of this review, we have focused on the 19 countries (Table 1) classified by the World Bank as from the Middle East and North Africa (MENA) region [14]. There are of course many differences between countries, but across this region the beliefs, cultures, and traditions of the people have many similarities. The literature suggests that both Arab and Muslim culture make professionals reluctant to withhold any intervention for patients in the ICU, and discussion of death and dying is often avoided by both families and healthcare professionals [15, 16]. Professional culture in ICUs is often described as hierarchical, with medical staff reserving the right to speak with families about EOL care and nurses reluctant to speak openly with families without medical approval [17]. Around 60-70% health care staff are from overseas, and so do not share patients’ culture, beliefs, or first language [18–20].

**Table 1:** Countries (19) classified by the World Bank as from the Middle East and North Africa MENA region

Algeria, Bahrain, Djibouti, Egypt, Iran, Iraq, Jordan, Kuwait, Lebanon, Libya, Morocco, Oman, Qatar, Saudi Arabia, Syrian Arab Republic, Tunisia, United Arab Emirates, Palestine, and Yemen.	

Middle Eastern countries have undergone rapid development of their healthcare systems, including the area of critical care [21]. However, palliative and EOL care has been much slower to develop [22]. Palliative care was first introduced to Middle Eastern countries in the early 1990s [23]. Despite this early introduction, access to palliative care is limited in most countries in the Middle East [24–26], with a significant shortage in palliative care programs compared to the high incidence of serious illness [27]. Even in Saudi Arabia, where there has been significant progress in introducing palliative and end-of-life care in the health care system, specialist services are not widely available, public knowledge is limited, and there is a persistent focus on curative treatments [28]. There is little consensus on practice regarding dying patients in Middle Eastern countries, as religious and cultural values raise additional challenges to the implementation of EOL care, especially where this may entail a ‘Do not resuscitate’ (DNR) decision [29, 30]. An assessment of knowledge, beliefs, obstacles, and resources available in the provision of palliative care services in fifteen Middle Eastern countries Silbermann et al. [31] found that major barriers include lack of palliative care beds and services, training for healthcare providers, community awareness, access to hospice services, and insufficient time and personnel.

Unsurprisingly, the general limitations of palliative and EOL care provision are mirrored in the critical care environment. Substituting palliative care for aggressive life prolonging therapy for dying patients in intensive care is a new concept with no legal definition for many healthcare providers in the Middle East. Consequently, there may be confusion in interpreting such terms as ‘not to be resuscitated’ and ‘comfort care’ among physicians [32]. There is a low prevalence of withdrawal of life-sustaining treatment in ICUs in many regions of the Middle East [33]. The most acceptable practice of limiting therapy for terminally ill patients in ICU involves avoiding escalation while continuing present therapy [32]. It was found that the dominant culture is to preserve life, providing maximum life-supporting interventions to all patients regardless patient’s condition. When patients approach EOL, the focus may shift to DNR decisions but not necessarily to providing palliative care and support for the patients’ families [32, 34].

Given the low prevalence of comprehensive palliative and EOL care offered in ICUs in Middle Eastern countries, it is important to understand the challenges to implementation (such as those discussed above) and how care can be supported and optimized [32].

### Research question

What helps or hinders effective end-of-life care in adult intensive care units in Middle Eastern countries?

## Methods

This systematic review is guided by the Preferred Reporting Items for Systematic Reviews and Meta-Analyses (PRISMA) guidelines, which were developed to ensure the transparency of data reporting in systematic reviews [35, 36].

### Study eligibility criteria

- Primary studies of palliative and end-of-life care in adult intensive care units in countries from the Middle East and North Africa (MENA) region (Table 1) as defined by the World Bank [14].
- English language studies were included.
- Reviews, reports, and guidelines were excluded.

### Information sources and search strategy

The research team identified primary studies from searching the following electronic databases: Medline, Embase, CINAHL, PsycInfo and Scopus. The search terms included: (End of life, OR death, OR dying OR terminally ill OR End-of-life care, OR terminal care, OR Palliative care) AND (Intensive care, OR critical care) AND (Algeria OR Bahrain OR Djibouti OR Egypt OR Iran OR Iraq OR Jordan OR Kuwait OR Lebanon OR Libya OR Morocco OR Oman OR Qatar OR Syrian Arab Republic OR Tunisia OR United Arab Emirates OR Palestine and Gaza OR Yemen OR Saudi Arabia OR Middle East OR Muslims OR Islam OR gulf states OR Arabs). In addition, we scanned the reference lists of the included studies. The team conducted the search in April 2022 and was not limited to a specific period.

### Study selection process

After removing duplicated articles, three researchers P.O, B.B and N.A independently screened study titles and then abstracts from all databases for eligibility. Following that, the team read and discussed the full texts of the remaining studies and resolved disagreements on eligibility.

### Data extraction

The researchers independently performed the data extraction and then checked for consistency to confirm accuracy of data reported. The following data were taken from each included study: author’s name, year of publication, objective, population and setting, study design and sample, measurement tool, main findings, and conclusions (Table 2) (Table 3).

**Table 2:** Summary of the included quantitative studies

First author/ year Country Objective	Population and Setting	Design and sample	Measurement tool	Findings	Conclusions
Mani (2017) Country: Saudi Arabia Objective: To explore nurses’ perceptions of obstacles to the provision of end-of-life care in the intensive care unit (ICU) in Saudi Arabia.	Conducted in a 936 bed specialist hospital in Riyadh between March and April 2015 There were 129 adult ICU beds in 6 specialist ICUs, including medical, hematological, oncological, surgical, and cardiac. All nurses working in ICU were eligible for the study.	Quantitative cross-sectional design Convenience sample of 77 ICU nurses	NSCCNR-EOLC Questionnaire Measures reported intensity and frequency of 29 obstacle items on a Likert scale ranging from 0–5. Intensity: 0 = least intense; 5 = most intense. Frequency: 0 = never occurs; 5 = always occurs. Open-ended questions: additional information on obstacles; aspects of care they would change; other comments.	Response rate was 62% (87/140). 10 incomplete responses were excluded. The mean score for each intensity items was calculated. Scores ranged from 1.27 to 4.26. The four highest obstacle intensity items were related to family issues, including Families not accepting what the physician is telling them about the patient’s poor prognosis. (4.26), The nurse having to deal with angry family members. (4.13) and family and friends who continually call the nurse wanting an update on the patient’s condition rather than calling the designated family member for information (4.06). These obstacles were also ranked in the top six for frequency	The major barriers were related to communication with and caring for patients’ families. Patient’s family, physicians with different opinions, cultural differences and language barriers were also highlighted. Nurses also reported the need for educational awareness and involvement of family in end-of-life care and futile care.
Almansour (2019) Country: Jordan Objective: To determine perceptions of Jordanian critical care staff about intensity and frequency of obstacles and facilitators to end-of-life care.	Conducted in two teaching hospitals and in five critical care units. The hospitals have western style health care. Critical care staff were eligible if they were involved in providing care for dying patients and employed in the units at the time of the study.	Quantitative Cross -sectional design Convenience sample of 104 ICU staff (76 nurses + 28 physicians) for Obstacles survey 76 ICU nurses for facilitators survey	NSCCNR-EoLC Questionnaire The first section uses a 5-point Likert scale to measure perceptions of intensity of 29 obstacles to EOL care ranging from 0 (not an obstacle) to 5 (an extremely large obstacle) and the frequency of their occurrence, ranging from 0 (never occurs) to 5 (always occurs). The second section uses a 5-point Likert scale to access perceptions about the intensity of 24 facilitators to EOL care, ranging from 0 (not a help) to 5 (extremely large) and the frequency of occurrence, ranging from 0 (never occurs) to 5 (always occurs).	The overall response rate was 72.7% (*n* = 104/143). 76 nurses (69.1%) and 28 physicians (84.5%) responded. Nurses and physicians perceived that the most intense obstacle to EOL care was: “family members not understanding what life-saving measures really mean” (Nurses: M = 4.12; Physicians: M = 3.92), then “clinicians who are evasive and avoid having conversations with family members” (Nurses: M = 3.71; Physicians: M = 3.46). The most intense facilitator to EOL care perceived by nurses was “having family members accept that the patient is dying” (M = 4.12).	Nurses and physicians agreed that the highest scoring obstacles were related to family members and the poor design of critical care units. Other highly scoring obstacles related to clinicians’ behaviours, characteristics and attitudes. Nurses perceived the highest scoring facilitator was related to family members and then the physicians practice/agreement about the care.
Attia (2013) Egypt Questions: Which barriers to providing EOL care to critically ill patients do critical care nurses perceive as the most intense? Which supportive behaviors to providing EOL care to critically ill patients do critical care nurses perceive of great help?	The study was conducted in four ICUs at Mansoura University Hospitals, Egypt, namely the oncology ICU, the coronary care unit, the hepatic ICU, and the surgical ICU.	Quantitative Cross-sectional design Convenience sample of 70 ICU nurses	The instrument adapted from NSCCNR-EOLC and translated into Arabic. 25 barrier items and 19 possible help behaviors. A 4-point Likert-type scale ranging from 1 = not a barriers/help to 4 = a great barrier/help.	Response rate 100%. The response to each items in the survey was calculated in percentage. The top items reported as severe barriers were associated with issues related to the ICU environment such as nurses’ heavy workload (81.4%), the poor ICU design (67.1%), and the liberal unit visiting hours (51.4%). Some items were related to patients’ family such as family members who do not understand the meaning of life-saving measures (65.7%) and family who continually call the nurse for updated information on the patient’s condition (62.9%). The highest supportive behaviors were nurses’ support involved good communication between physicians and nurses caring for the dying patient (94.3%), nurses drawing on their own previous experiences (82.9%), and supporting each other after the death of their patients (75.7%)	Barriers to providing EOL care were mainly related to intensive care environment, family members, followed by nurses’ knowledge and skills, physicians’ attitudes and treatment policy. However, the highest possible help to providing EOL care were nurses’ support and family-centered care, and families’ support.

**Table 3:** Summary of the included qualitative studies

Author/ Year Country Objective	Population and setting	Design and sample	Findings	Conclusions
Al Mutair (2020) Saudi Arabi Objective: To identify the needs, beliefs, and practices of Muslim family members during end-of-life care for a family member in the intensive care unit (ICU) in Saudi Arabia.	Conducted in the ICU of a 320-bed tertiary referral hospital in Dahran city in Saudi Arabia. The ICU has 36 beds including 14 neonatal beds, four adult post-cardiac surgery beds, eight coronary care beds, and 10 general adult ICU beds. However, only family members of adult patients were interviewed.	Qualitative a phenomenological study In-depth interviews conducted with 10 Family members of dying patient in ICU between September 2016 and March 2017.	The four major themes were: (a) the spirituality of death, (b) family’s need for information, (c) being there for enough time, and (d) having good space at bedside.	Participants placed high value on religious practices such as prayer, and appreciated when these practices could be accommodated in the ICU. They also detailed their need for frequent communication about the patient condition.
O’Neill (2017) Bahrain Objective: To explore nurses’ care practices at the end of life, with the objective of describing and identifying end of life care practices that nurses contribute to, with an emphasis on culture, religious experiences and professional identity.	It was conducted in the two ICUs of two hospitals that are the main providers of acute care.	Qualitative -Grounded theory Semi-structured in-depth interviews with 10 ICU nurses (five from each ICU).	A core category, Death Avoidance Talk, emerged. This was supported by two major categories: (1) order-oriented (medically directed) care: nurses were consulted by medical staff but not involved in decisions; and (2) signalling death and shifting the focus of care to family members. The organisation was hierarchical, with nurses deferring to doctors in end-of-life discussions with families. Yet medical staff were reluctant to speak plainly about death with families. Consequently, communication was unclear, treatments prolonged, and death sometimes unexpected by families. Nevertheless, there were feelings of respect and compassion towards the families.	Despite the avoidance of death talk and nurses’ lack of professional autonomy, they created awareness that death was imminent to family members and ensured that end of life care was given in a culturally sensitive manner and aligned to Islamic values. Of all the nurses interviewed, none had received any specialist education in ethics or palliative care. Specialist education and training is needed.
Abu-El-Noor (2016) Palestine Objective: to examine how Palestinian nurses working in intensive care units (ICUs) understand spirituality and the provision of spiritual care at the end of life.	It was conducted at the two major hospitals in Gaza Strip, Palestine. The first hospital had 740 beds, of which 12 were ICU beds, and 24 ICU nurses. The second hospital had 240 beds including 12 ICU beds and a total of 18 ICU nurses. Gaza Strip has five ICUs with a total of 39 beds and 89 ICU nurses.	Qualitative Semi-structured in-depth interviews 13 ICU nurses	The following themes were identified: meaning of spirituality and spiritual care, identifying spiritual needs, and taking actions to meet spiritual needs. Spirituality was mostly thought of as expressing Islamic religious needs and practices. Spiritual needs were identified by talking with family members (and sometimes patients) and by assessing how close patients were to death. Actions included shifting the goals of care to comforting; allowing more visiting; reciting the Quran; enabling prayer.	Most of the spiritual care provided was based on religious beliefs and practices, thus illustrating the importance of the role of religion in providing healthcare. Nurses used both communication and observation to identify spiritual needs of patients and provide relevant spiritual care. It was recommended to increase the emphasis on the provision of spiritual care for all patients.
Borhani (2014) Iran Objective: to explore intensive care nurses’ perspectives of the end-of-life care in an Islamic context in South-east of Iran	It was conducted in three ICUs at an Iranian teaching hospital affiliated to Kerman University.	Qualitative semi-structured interview 12 ICU nurses	Four major categories emerged from analysis of the interviews: commitment to care, awareness of dying patients, caring relationships, and dealing with barriers and ethical issues. The ICU nurses emphasis on creating a spiritual caring environment to enable patients, families and even nurses to achieve a spiritual comfort. Physical care of dying patients may not be useful in their cure but is a prerequisite of spiritual care causing families and nurses to become satisfied. Nursing thinking is restoration and resuscitation, and futile care is prohibited in nursing on the care of people in any conditions and times. Care is never futile, but medical interventions sometimes are.	The first category commitment to care, was emphasized and appeared dominant in all interviews. It was concluded that emphasis on creating a spiritual caring environment is needed to enable patients, families and even nurses to achieve a spiritual comfort.
Hamdan Alshehri (2021) Saudi Arabia Objective: to explore the association of organisational structures when integrating palliative care in intensive care units.	The data were collected by conducting interviews between April and July 2019, at four Ministry of Health hospitals in Riyadh, Saudi Arabia; in two tertiary referral specialist hospitals and two secondary general hospitals.	Qualitative descriptive/ in-depth interviews 15 managers and 36 health care professionals working in intensive care	Three themes were identified: Do not resuscitate policy as a gateway to palliative care, facilitating family members to enable participation and support and barriers for palliative care in intensive care unit as a result of intensive care organisation. Both managers and health care professionals working closely with patients in ICU pointed to the organisational structures as a major block in integrating a palliative care approach into intensive care situations. The lack of palliative care policy in intensive care opened up spaces in which moral dilemmas were confronted including do not resuscitate policies and practices and especially those dilemmas related to personal beliefs influenced by religion and culture. This may create barriers for the integration of palliative care in ICU.	The findings indicate the need for specific palliative care policies and implementation strategies tailored according to practice needs.
Alasiry (2012) Saudi Arabia Objective: to explore the nurses’ experiences of providing palliative care for critically ill patients in an intensive care unit in Saudi Arabia.	intensive care unit in Saudi Arabia, it included Medical- Surgical ICU and long-term ICU.	Qualitative, semi-structured interview with 9 ICU nurses	The study highlights the important aspects of palliative care e.g. symptoms control, communication, team work and family support Six themes were identified: Care in the ICU is challenging; Collaborative work to achieve patient’s needs; Caring as a holistic approach; experiencing language as a support; experiencing language as a barrier; and Family-patient centered care and support. The majority of nurses in the study are non-Arabic speakers and they found that language is a barrier to communicate with a patient and family. However, different protocols was available to standardized care to deal with different symptoms, in addition to having competencies that keep them updated to achieve maximum patient care.	Communication was a barrier when non-Arabic speaking nurses provide palliative care for critically ill patients and their families. Therefore, the patient and family’s involvement and the spiritual care appears insufficient in this ICU.

### Study assessment process

The team assessed the methodological quality of all included studies using the Joanna Briggs Institute (JBI) critical appraisal tools [37]. We calculated quality scores by totaling the number of ‘yes’ responses to criteria in the critical appraisal checklist. Quantitative cross-sectional design studies could achieve a maximum 8 points, and qualitative studies 10 points.

### Data synthesis

The heterogeneity of study designs and outcomes precluded meta-analysis, so we carried out a narrative synthesis. We summarized narratively in text the data extracted from studies and presented them in tabular form. Then, the team sorted the findings into five thematic categories according to their common characteristic with the frequency of studies presented. Subsequently, the team summarised the included studies in a narrative synthesis, which was drafted by one author and reviewed, and validated independently by the research team members [38, 39].

## Results

### Study selection

**Fig. 1:**
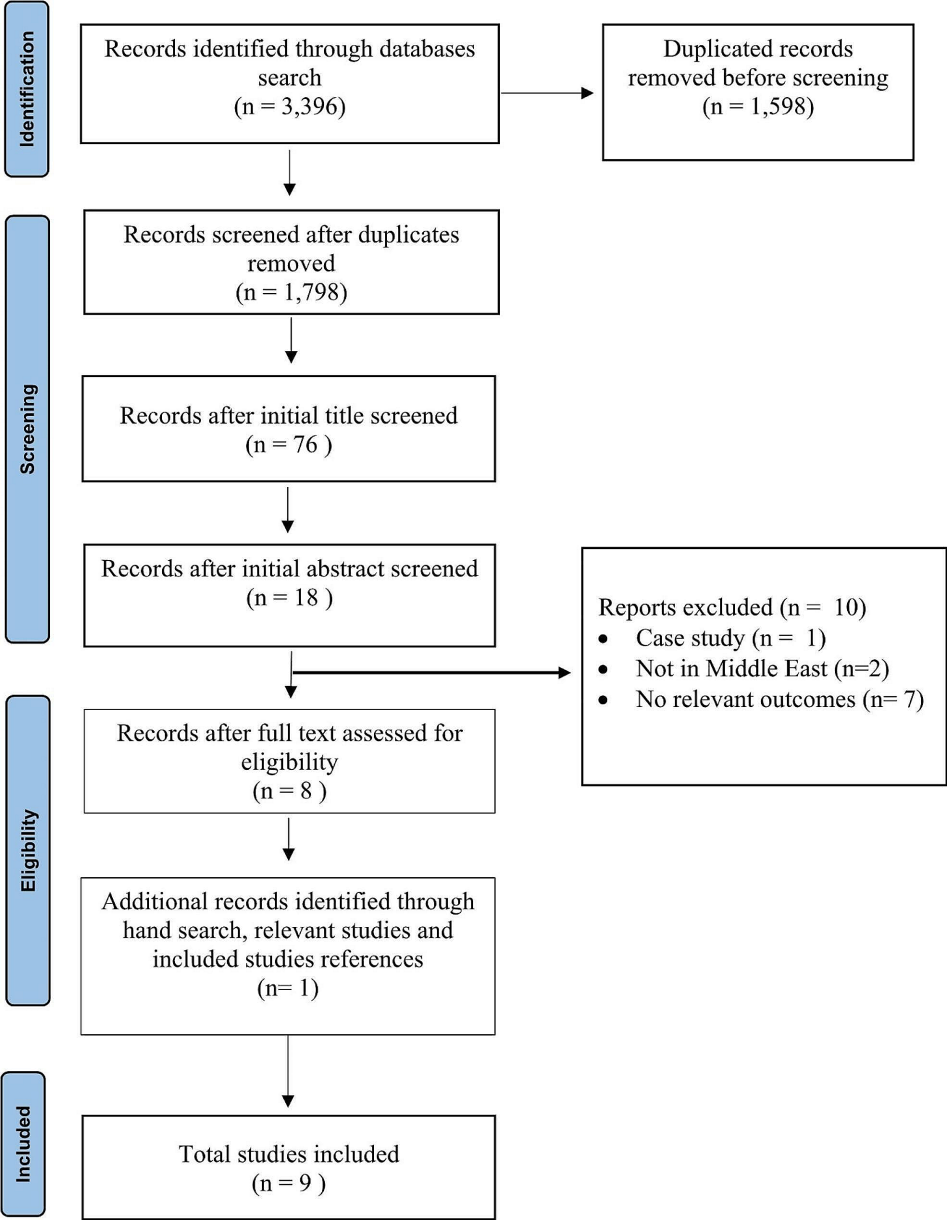
PRISMA flowchart diagram

The initial search of databases produced 3,396 documents (Fig. 1). After removing duplicated studies, we screened 1,798 titles for eligibility from all databases. After initial screening of titles, we screened 76, and excluded 58 studies. Researchers read the remaining 18 full text studies and excluded 10 studies as they did not meet the eligibility criteria: one study was a case study, two studies were not conducted in a Middle Eastern country, and seven studies were assessed not to have relevant outcomes. We added one additional study, which had been identified from screening relevant review and reference lists of included studies. Therefore, nine studies are included in this review (Fig. 1). We had planned to exclude papers not written in English but in fact all papers identified through our search were written in English.

### Study characteristics

#### Study designs

Of the nine included studies, three were quantitative, cross-sectional studies (Table 2), and six were qualitative studies (Table 3).

### Methodological quality

The six qualitative studies were classified as measured five and above out of eight [40–45]; and the three quantitative cross-sectional studies measured eight and above out of 10 [46–48]. using the JBI) critical appraisal tools (Table 4) (Table 5).

**Table 4:** JBI assessment for Quantitative studies

Author/ year	1 Were the criteria for inclusion in the sample clearly defined?	2 Were the study subjects and the setting described in detail?	3 Was the exposure measured in a valid and reliable way?	4 Were objective, standard criteria used for measurement of the condition?	5 Were confounding factors identified?	6 Were strategies to deal with confounding factors stated?	7 Were the outcomes measured in a valid and reliable way?	8 Was appropriate statistical analysis used?	Score
[46]	Yes	Yes	Yes	Not clear	NA	NA	Yes	Yes	5
[47]	Yes	Yes	Yes	Not clear	NA	NA	Yes	Yes	5
[48]	Yes	Yes	Yes	Not clear	NA	NA	Yes	Yes .	5

**Table 5:** JBI assessment for Qualitative studies

Author/ year	1 Is there congruity between the stated philosophical perspective and the research methodology?	2 Is there congruity between the research methodology and the research question or objectives?	3 Is there congruity between the research methodology and the methods used to collect data?	4 Is there congruity between the research methodology and the representation and analysis of data?	5 Is there congruity between the research methodology and the interpretation of results?	6 Is there a statement locating the researcher culturally or theoretically?	7 Is the influence of the researcher on the research, and vice- versa, addressed?	8 Are participants, and their voices, adequately represented?	9 Is the research ethical according to current criteria or, for recent studies, and is there evidence of ethical approval by an appropriate body?	10 Do the conclusions drawn in the research report flow from the analysis, or interpretation, of the data?	Score
[40]	Yes	Yes	Yes	Yes .	Yes	Yes .	No	Yes	Yes	Yes	9
[41]	Yes	Yes	Yes	Yes	Yes	not clear .	No	Yes	Yes	Yes .	8
[42]	Yes	Yes	Yes	Yes	Yes	not clear .	No	Yes	Yes	Yes	8
[43]	Yes	Yes	Yes	Yes	Yes	Not clear	No	Yes	Yes	Yes	8
[44]	Yes	Yes	Yes	Yes	Yes	Yes	Not clear	Yes	Yes	Yes	8
[45]	Yes	Yes	Yes	Yes	Yes	Not clear	Not clear	Yes	Yes	Yes	8

### Main objectives of the studies

Cross-sectional studies sought to measure ICU staff perceptions of barriers and supportive behaviors to the provision of end-of-life care in the intensive care unit [46–48]. The qualitative studies focus on exploring critical care nurses’ experiences and perceptions of providing end-of-life care for critically ill patients in an intensive care unit [43, 45]; evaluating nurses’ care practices at the end of life and their understanding of the provision of spiritual care at the end of life [41, 42]; identifying the needs, beliefs, and practices of Muslim family members during end-of-life care for a family member in the intensive care unit [40]; and the impact of organisational structures when integrating palliative care in intensive care units [44].

### Study populations

With the exception of one study that focused on family members [40], all the studies were of healthcare professionals. Participants in six studies of the studies were ICU nurses [41–43, 45, 46, 48]; one study involved ICU managers alongside health professionals [44] and another study involved ICU physicians and nurses [47].

### Cultural context

The findings of this review can only be fully understood in the light of the cultural and religious context of MENA countries, and the related professional cultures in healthcare. Both Arab and Muslim culture make professionals reluctant to withhold any intervention or medication for any patient in the ICU, and Muslim culture and practices (such as reading the Qur’an to the patient, performing prayer, placing the dying patient to face toward Mecca and saying the *Shahadatain* or testimony of faith) are often important to families. God is seen as the ultimate healer, who is able to heal the sick person at any stage, which feeds into a desire to keep the patient alive at all costs. Consequently, while some families are open about their loved one’s likely prognosis, discussion of death and dying is often avoided by both families and health care professionals [40], and withdrawing non-effective treatment may be seen as complicity in causing the patient’s death [44]. The patient’s death may not be faced until medical staff make a DNR decision and even this is sometimes opposed by families, which further limits discussion of EOL, and so curtails the EOL care offered to both patient and family.

Communication with the family and their involvement in decisions about the patient can vary greatly between ICUs in different regions [41, 44]. However, the professional culture in ICUs is often described as hierarchical, with medical staff reserving the right to speak with families about EOL care and yet often showing reluctance to make a DNR decision or to withdraw ineffective treatments. Nurses do not consider themselves professionally autonomous in these situations, and so are reluctant to speak openly with families without medical approval, resorting to hints and veiled references [41]. These difficulties in communicating with families are compounded by language barriers, in that many health care staff are from overseas [45]. Frequently, they do not share patients’ culture and beliefs, and usually speak English rather than Arabic, whilst Arabic is the first language for most families [41, 45, 46].

### End-of-life care challenges and supportive behaviours

Within the overall cultural context described above, five main themes were identified as EOL care challenges and/or facilitators: organisational structure and management, (mis)understanding of end-of life care, spirituality and religious practices for the dying, communication about EOL care, and the impact of the ICU environment.

### Organisational structure and management

Many ICUs lack policy and guideline documents in relation to palliative care. This leaves health care professionals without clear guidance and support in relation to DNR decisions and other clinical and ethical dilemmas. This means health care professionals are thrown back on their personal beliefs and cultural assumptions, as described above. This may create barriers for the integration of palliative care in ICU [44]. As noted above organisationally, ICUs may be hierarchical, with nurses deferring to doctors in end-of-life discussions with families, and a lack of professional autonomy and involvement in decision making. Yet medical staff were reluctant to speak plainly about death with families. Consequently, communication was unclear, treatments prolonged, and death sometimes unexpected by families. In addition, nurses identified a need for specialist education and training but reported none had received training in ethics or palliative care [41].

### (Mis)understanding of end-of life care

The most reported challenge across the studies was related to different understandings of EOL care between healthcare team and family members [46–48]. Families may believe that introducing EOL care means actively ending the patient’s life, rather than providing the best supportive care and comfort measures when death has become inevitable. On the other hand, medical staff may avoid discussion with families about the patient’s prognosis and related EOL care, which contributes to the confusion for families and may lead to them not accepting the patient’s poor prognosis and misunderstanding what life-saving measures really mean. Some relatives may think that EOL care means abandoning the patient and neglecting ordinary care and treatment; or not appreciating how traumatic interventions such as CPR may be for the patient [46–48].

On the other hand, EOL care is facilitated when ICU staff take active steps towards patient and family-centred care. Family members can be taught how to approach caring for their dying relative. Agreeing a specific family member as the main communication link with the family enabled better communication of goals of care for the dying patient and avoided misunderstanding [48].

If family members are helped to accept that the patient is dying, this allows ICU staff to discuss EOL care and also provide appropriate support for the family, for example by providing a peaceful, dignified bedside scene for family members as their loved one approaches death, and by having the physician meet in person with the family after the patient’s death to offer support and reassure them that all possible care was given [47].

### Spirituality and religious practices for the dying

Family members of critically ill patients place a high value on the spirituality of death, and religious practices such as prayer, and appreciate it when these practices can be accommodated in the ICU. However, family members may believe that God is the ultimate healer, and so hold on to hope of recovery in the face of a poor prognosis, or even deny the possibility of death [40]. This presents a challenge to physicians who may wish to discuss EOL care and DNR decisions with family members [47]. On the other hand, nurses are capable of delivering end-of-life care in a culturally sensitive manner and aligned to Islamic values [41]. Engagement with family members (and sometimes patients) about spiritual needs when facing EOL allowed nurses to facilitate spiritual care, usually based on religious beliefs and practices [42]. Spiritual and physical care may overlap, as keeping the patient clean, having someone recite the Holy Qur’an to the patient, closing the person’s eyes as they die, and after death aligning the body towards Mecca, are all part of asking God to comfort, forgive, or heal the individual. Carrying out these practices may provide spiritual comfort to both families and nurses [43]. However the spiritual care appears insufficient in some ICUs due to diversity of beliefs among nurses [45].

### Communication about EOL care

Family members of ICU patients suggested that the need for frequent updates about the health status and prognosis of their loved one was an important part of end-of-life care [40]. However, the majority of ICU nurses are non-Arabic speakers as a result they found it difficult to communicate with patients and their families about EOL care [45].

Family members of ICU patients suggested that nominating a particular member of the medical member to provide information during visiting hours aided communication, as did the presence of social worker. The input of a social worker is important when communicating bad news and clarifying complex issues around EOL care. In addition, Attia [48] found that 94.3% of critical care nurses report effective communication between physicians and nurses caring for the dying patient as facilitator. This can ensure the staff are updated about that all possible care was provided and that family support was given by a multi-professional team.

#### Impact of the ICU environment

Other frequently identified obstacles to EOL care, related to the ICU environment reported by health professionals include poor design of critical care units, restricted visiting hours, and nurses’ heavy workload [48]. It was noted that the ICU bedside of patient does not allow for privacy of dying patients or grieving of family members. Therefore, it was difficult for family members to stay as much as they wish close to the dying patient. Likewise, critical care nurses report that providing a peaceful, dignified bedside scene for family members to grieve in private helped facilitate EOL care for the family [47, 48]. It is also the case that nursing workload limits the time to provide the family with needed emotional support, which was reported as a further burden on nurses [40, 47].

## Discussion

Only nine studies were included in the review, all observational rather than intervention studies, and six of them qualitative. Studies were moderately strong methodologically. The qualitative studies provide useful insights on the perceptions, experiences, attitudes and behaviors of healthcare professionals and patients’ families in relation to end-of-life care; while the three quantitative studies more directly measured the barriers and facilitators of EOL care in different middle eastern counties.

We found common cultural characteristics across the studies which had a profound effect on the perception and delivery of EOL care. The natural desire and expectation of families – and of health care professionals - is that health care is directed to the healing and restoration of their loved one, especially in the highly technological and interventionist context of the ICU. This, coupled with the hope that God will intervene to heal the critically ill person, seems to produce a social and psychological barrier to discussions of EOL care and the withdrawal of treatments that have become ineffective. These discussions and actions may be seen as synonymous with abandoning the patient or even actively ending their life.

In this overall cultural context, five interlocking phenomena that helped or hindered EOL care in ICU were identified: organisational structure and management, (mis)understanding of end-of life care, spirituality and religious practices for the dying, communication about EOL care, and the impact of the ICU environment. These themes were often mutually reinforcing in providing barriers to effective EOL care. For example, a hierarchical organisational structure that disempowers nurses in relation to communicating with families, is exacerbated by language barriers and the limitations of the ICU environment. Similarly, misunderstandings about the goals of EOL care may be compounded by the language and cultural distance between health care professionals, and patients and their families.

This is not an argument for marginalizing the cultural and religious concerns of patients and their families. There is evidence from the literature that where nurses deliver end-of-life care in a culturally sensitive manner and aligned to local Islamic and Arabic values, and make room for spiritual and religious practices, then communication can be effective, and all concerned can benefit from these spiritual and cultural resources [32, 34, 49].

In relation to organisational structure and management, the review identified a lack of guidance around EOL care contributing to uncertainty for ICU staff. Guidance is available [50], and the literature suggests organisations should have administrative processes in place to encourage critical care teams to lead the discussions and begin early EOL care plans when appropriate [6, 51]. In addition, the application of a designed checklist and reminder to discuss end-of-life aspects may allow physicians to evaluate the situation and decide whether there is a need for palliative care consultation [52–54]. The interprofessional tensions between nurses and doctors reported in this review are common in ICUs, including in EOL care situations [17]. Hierarchical relations with senior doctors can contribute to moral distress for more junior staff and to unnecessary and burdensome treatment for patients [55]. The ICU is a complex social context, with multiple interactions between patients, their families and the various professional groups responsible for care [56]. Addressing the limitations of interprofessional working will require long-term engagement with stakeholders and an understanding of the cultural context for care [57]. As a minimum, the review has made clear the need for investment at an organisational level in appropriate education and training for ICU staff in order to promote interprofessional collaboration, and effective engagement with patients and their families in relation to EOL care.

Differing understandings in relation to end-of life care between families and ICU staff – compounded by poor communication - can lead to conflict and loss of trust. Family members experiencing difficulty in accepting a poor prognosis and resisting withdrawal of ineffective treatments is not unique to MENA countries. Similar challenges have been reported in Australian [51] and Canadian [52] contexts, where it was reported that the largest barrier to transition to end-of-life care was unrealistic family expectations. Gries et al. [58] acknowledge the importance of involving and supporting the patients’ family in EOL decisions. In these circumstances it is recommended that family members are provided with the goals of care to help them navigate decisions around EOL care [52, 59]. This involvement is thought to lead to a better understanding of the treatment plan as well as enabling family members to have more realistic expectations regarding the patient’s prognosis and the effectiveness of treatment [52]. Family meetings are considered an effective approach to ensure patient- and family-centred care in palliative care. Family engagement in a serious illness discussion can clarify the values of patients and relatives, provide information, determine care preferences, and identify sources of illness-related distress. Among other benefits, these interventions may reduce family distress, mitigate unmet needs, prepare families, and improve bereavement outcomes. Palliative care experts believe that family meetings can reinforce the therapeutic alliance with families, facilitate consensus, and enhance families’ understanding of the patient’s serious condition [60].

Spiritual practices were given a high value by family members of ICU patients at end of life. This is a complex area of care, where misunderstanding may impede EOL care but sensitive engagement with the family can be very helpful. Some religious and spiritual beliefs can make discussions of EOL care challenging for all parties. On the other hand, spiritual and physical care may helpfully overlap, and consistent with the work of Kisorio and Langley [61], most families valued prayers and religious support during this difficult time. Spirituality is considered as a source of hope, and when prognosis is poor, as a means of seeking comfort, coping, and allowing the patient to die peacefully in a dignified manner. In this respect MENA countries may well be in a stronger position than others, as spiritual support is reported as lacking [61, 62].

Reported challenges in communication about EOL care are consistent with research in South Africa [61] and the United Kingdom [63], which found that clear communication and receiving relevant information about the patient’s progress on a regular basis was one of the important needs raised by family members. This review highlighted the value of having an identified family member and staff contact to facilitate communication. This aligns with previous research reporting inadequate communication and inconsistencies in information received from different physicians [64, 65]. Festic et al. [66] reported that improving communication among ICU team and with families was identified by 30% of the respondents as the change most required to improve EOL care. Similarly, Curtis et al. [67] emphasized that effective communication between healthcare professionals and families improves family understanding, clinical decision making and psychological well-being of family members.

Language and cultural barriers to communication have also been reported in the wider literature. Barwise et al. [68] noted that in the context of an unfamiliar language and culture, some physicians lack the skills necessary for adequate and effective discussions and may fear miscommunicating. As result, infrequent communication leads to patient and family distress, mistrust, and inadvertent misconceptions. In this situation, availability of interpreters in the organisation and specifically in ICU is an important support that facilitates communication and discussion of end-of-life care [69].

Families and healthcare professionals report that the ICU environment was not convenient for EOL care due to lack of private space and limited visiting time. Families need to be beside their dying patient and the poor design of ICUs makes it difficult for relatives to stay with the patient as much as they wish. These results agreed with the findings of Millner et al. [70] and Kyeremanteng et al. [52] who conclude that the lack of privacy could hinder the provision of quality end-of-life care. Limited visiting hours for dying patients in ICU was also viewed as a barrier to providing end-of-life care. Liberal flexible visiting hours should be considered for family based on the patient’s condition. Ghiyasvandian et al. [71] showed that most nurses believed that family presence with the patient can improve emotional support at end of life. This was supported by Hodde et al. [72], who suggested that ICU patients have a better quality of dying if they do not die alone. However, ICU visiting hours continue to be restricted [73].

### Strengths and limitations

We used a broad search strategy that reduced the risk of missing relevant studies, and performed study selection, data extraction, and data synthesis in duplicate. However, most of the included studies focus on critical care nurses’ perceptions, which indicates that the review could not effectively represent the challenges and facilitators experienced by the multidisciplinary team involved in the delivery of end-of-life care in the ICU settings. The studies were drawn from only six of 19 MENA countries, and so may not reflect practice in other countries. Given the focus of the papers, many important palliative care practice (such as symptom control and family interventions) are not discussed as part of the review. In addition, this review is limited by inclusion of English language papers only.

### Conclusion and recommendations

This review identified five phenomena influencing the quality of EOL care in adult intensive care units in MENA countries: organisational structure and management, (mis)understanding of end-of life care, spirituality and religious practices for the dying, communication about EOL care, and the impact of the ICU environment. These are certainly not unique to the Middle East but can be found throughout the international literature. However, the cultural context of MENA countries gives these areas of practice special challenges and opportunities. Of course, all ICU care takes place in a particular cultural context, and patients, families, and ICU staff – whether in MENA countries or elsewhere – are likely to carry with them the assumptions and values of the surrounding culture. However, one unusual factor in many MENA countries is the large proportion (60-70%) of expatriates delivering care. These professionals may not share local cultural assumptions and also experience language barriers, which will make communication about EOL care challenging.

With these challenges in mind, we offer the following recommendations.

- Organisations should agree and make available suitable policies and guidance to provide a supportive framework for EOL care and reduce uncertainty and ambiguity for ICU staff.
- ICU staff should have access to appropriate education and training to promote interprofessional collaboration, and effective engagement with patients and their families in relation to EOL care.
- Where possible a named family member should be identified as the key communicator for the family and a member of ICU staff be similarly identified, with the aim of understanding the needs and perspectives of family members, avoiding misunderstanding, and effectively communicating goals of care, changes in prognosis, and reasons for treatment decisions.
- The value of spiritual and religious care should be recognized by ICU staff and end-of-life care delivered in a culturally sensitive manner and, where appropriate to the patient and family, aligned to Islamic and Arabic values.
- Where ICU staff are not confident in local languages, the organisation should make interpreters available to facilitate discussion of EOL decisions with the patient and family.
- The limitations of the ICU environment should be considered when the patient is nearing the end of life, with consideration given to longer and more flexible visiting hours, and providing privacy at the patient’s bedside.

Given the complex and interactive effects of these important characteristics, it appears that interventions to promote more effective EOL care in the ICU should seek to address the full range of issues, rather than target isolated aspects of care. Finally, we recommend further observational research to confirm or modify the results of this review, and with a view to developing and evaluating comprehensive interventions to promote effective EOL care in ICUs in the Middle East.

## Data Availability

All data is available in the manuscript.
